# Investigation of Cell-Substrate Adhesion Properties of Living Chondrocyte by Measuring Adhesive Shear Force and Detachment Using AFM and Inverse FEA

**DOI:** 10.1038/srep38059

**Published:** 2016-11-28

**Authors:** Trung Dung Nguyen, YuanTong Gu

**Affiliations:** 1 School of Chemistry, Physics and Mechanical Engineering, Science and Engineering Faculty, Queensland University of Technology, Brisbane, Queensland, Australia

## Abstract

It is well-known that cell adhesion is important in many biological processes such as cell migration and proliferation. A better understanding of the cell adhesion process will shed insight into these cellular biological responses as well as cell adhesion-related diseases treatment. However, there is little research which has attempted to investigate the process of cell adhesion and its mechanism. Thus, this paper aims to study the time-dependent adhesion properties of single living chondrocytes using an advanced coupled experimental-numerical approach. Atomic Force Microscopy (AFM) tips will be used to apply lateral forces to detach chondrocytes that are seeded for three different periods. An advanced Finite Element Analysis (FEA) model combining porohyperelastic (PHE) constitutive model and cohesive zone formulation is developed to explore the mechanism of adhesion. The results revealed that the cells can resist normal traction better than tangential traction in the beginning of adhesion. This is when the cell adhesion molecules establish early attachment to the substrates. After that when the cells are spreading, stress fiber bundles generate tangential traction on the substrate to form strong adhesion. Both simulation and experimental results agree well with each other, providing a powerful tool to study the cellular adhesion process.

Cartilage is the flexible connective tissue found in many parts of human and animal bodies such as nose, ear, elbow and knee. Articular cartilage is the hyaline and avascular tissue that covers the surfaces of the joints. Osteoarthritis is a type of joint disease which results in significant patient suffering and a large financial burden on both the health system and employers. Chondrocytes are cytoskeleton (CSK)-rich eukaryotic cells which are the mature cells in cartilage tissues and perform a number of functions within the tissue. It is well-known that the mechanical properties of these cells are significantly altered in the development and progression of osteoarthritis^1^,^2^,^3^. Moreover, it has been reported that the disruption of the collagen network in the early stages of osteoarthritis causes an increase in water content of the cartilage, which in turn leads to a reduction of the pericellular osmolality of the chondrocytes^4^. One of the common treatments for osteoarthritis is to replace damaged or diseased cartilage with artificial biomaterials. Biocompatibility, fabrication robustness and efficiency are the necessary requirements for these materials. These materials will also need to enhance cell adhesion ability.

Cell adhesion is important in many biological processes including cell proliferation, fate and migration^5^,^6^. It is well-accepted that the adhesive strength of cells varies with different substratum, materials, topography^7^,^8^,^9^ and chemo-mechanical properties of the surrounding cellular microenvironment^10^. In particular, the extracellular matrix’s molecular composition and mechanical properties have a pivotal role in cell spreading and migration^6^,^11^,^12^. Understanding the cell adhesive properties is crucial to tissue engineering^13^ which seeks treatments to the damaged or diseased tissues and organs through the replacement of combinations of cells, scaffolds and soluble mediators^14^. This approach needs readily available stem cell sources that can provide the relevant properties and behaviour under controlled conditions. Increasing evidence has demonstrated that cellular micro-environment plays an important role in controlling the activities and behaviours of stem cells. For instance, the stiffness of the substrate defined the differentiation lineage of the mesenchymal stem cells (MSCs)^5^. Therefore, a better understanding of cell adhesive behaviours would open insight into the fundamental bases between cells and the tissues. The knowledge will allow development of advanced technologies to improve tissue engineering and stem cell technologies.

Atomic Force Microscopy (AFM) provides a method to study the nanomechanical properties of biological tissues and explore the cell responses to external stimuli^15^,^16^,^17^,^18^,^19^,^20^,^21^,^22^,^23^. Besides being used to conduct the nano-scale mechanical test, AFM has also been used to measure the adhesion force of cells on different biomaterial surfaces^24^,^25^,^26^. There are three different strategies to measure adhesion force using AFM investigated in the literature (see Fig. 1). Among these, the last strategy utilized the AFM cantilever to apply a shear force to detach the cells in order to study the adhesive forces between cells and the substrate. This developed technique would provide a powerful tool to investigate the adhesive behaviour of chondrocytes. The advantage of this technique is that it utilizes the contact scanning mode that is available in any AFM system compared to other techniques that may require some special facilities^27^.

While a number of experimental techniques have been developed and applied in biomechanics studies, they still do not provide a comprehensive analysis of a single cell’s response to short- and long-term mechanical loads. This is due to the difficulties associated with conducting experiments on living cells in the real biological environment. In addition, many of the experiments cannot show the dynamic responses of a cell during a mechanical event. Numerical simulations, however, offer a way to gain insight into the biological and biomechanical processes of a cell under mechanical stimuli, as well as to calibrate model parameters and identify appropriate model assumptions. Finite Element Analysis (FEA), which is one of the most commonly used numerical simulation methods, is widely used to study the mechanical properties and behaviour of tissues, single cells and other components of a tissue. Therefore, a FEA model will be developed in this paper to study the dynamic processes of cells’ detachment under mechanical shearing stresses. The model will help us to have better understanding of detachment processes.

Consequently, this study will utilise AFM to measure the time-dependent adhesive properties of living chondrocytes. The experiments will be conducted by applying a shear force on the cells until they are detached. Details of this technique were already described in the literature^28^,^29^ (see Fig. 1c). The FEA model coupled with the porohyperelastic (PHE) mechanical model^30^,^31^ and a cohesive zone formulation^32^ will be used to explore the mechanism underlying the adhesive behaviour of single living cells. The PHE model is used in this study because it has been proven to be a powerful model to investigate the fluid-solid interaction within the cells^30^,^31^,^33^. The model proposed in this study is the first one to integrate multi-phase model with cohesive zone formulation to investigate the adhesive properties of chondrocytes. The model has potential to establish a novel numerical modelling method to predict early osteoarthritis and other diseases. This study will provide new knowledge about cell adhesive behaviour. In addition, this project has the potential in studying the early stage of osteoarthritis and other diseases that may be related to the change of adhesive properties of cells.

## Results and Discussion

### AFM lateral force detachment experiment

#### Cantilever detection limit

As presented by Zhang *et al*.^29^, before conducting AFM adhesion measurements, the detection limit of the applied cantilever needs to be identified. The reason is that if the detection limit of the cantilever is larger than the measured value, the measurement results might possess significant error. The principle is to use the setpoint value *V*_*setpoint*_, which is as small as possible but large enough to obtain good scanning image quality to calculate the cantilever’s detection limit using Eq. (5). This setpoint was chosen to be 0.3 V, which is the same as Zhang *et al*.’s value. The calculated detection limit of applied cantilevers in this study was around 2.0632 and 58.5746 nN for SHOCONG and ACSTG cantilevers, respectively (see Table 1). This limit is much smaller than our measured detachment forces of living cells in this study, which will be presented below, therefore the detection limit will not significantly influence the measured forces.

#### Measurement of lateral detachment force by AFM

**Figure 2:**
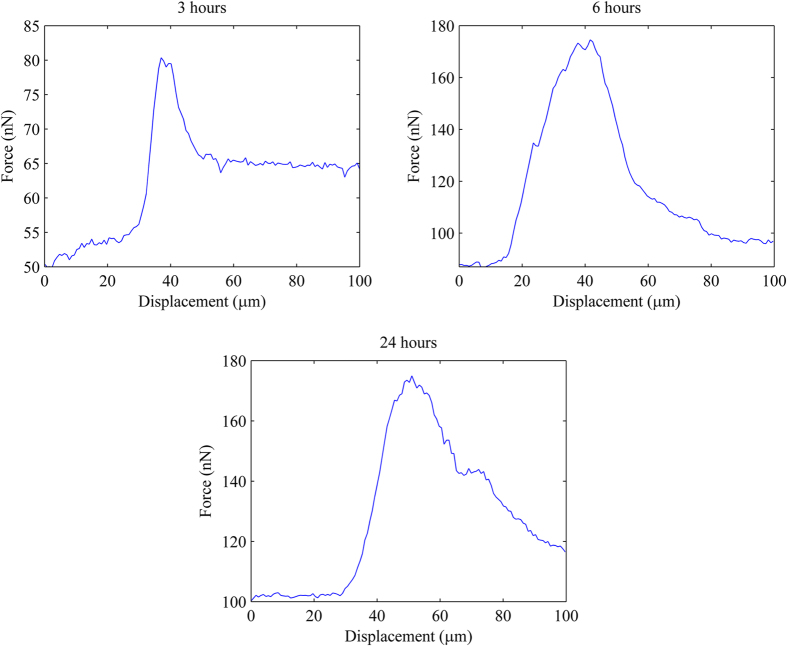
Typical curves of lateral detachment force versus displacement of chondrocytes seeded for 3, 6 and 24 hours.

**Figure 3:**
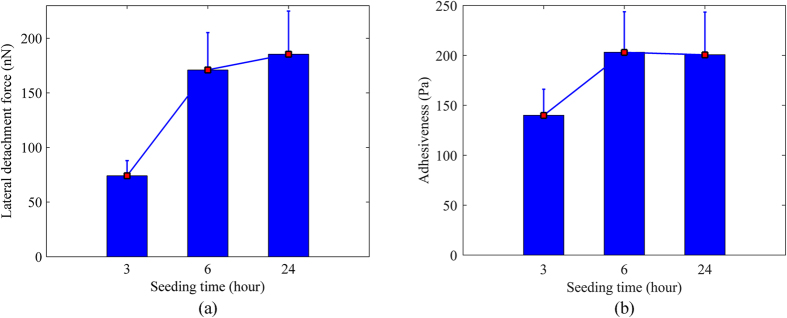
(**a**) Lateral detachment force and (**b**) mechanical adhesiveness of chondrocytes seeded for 3, 6 and 24 hours.

In previous works^28^,^29^, the AFM tip was used to repeatedly scan over the cells until they were detached. The disadvantage of this technique is that the adhesion properties of the cells might have been altered while being scanned. Therefore, in this study, the AFM cantilever was quickly moved to the middle of cells during scanning process to detach them with only one scan line. This technique would give more reliable and precise results. Note that the scan speed used in this study was 50 μm/s. Figure 2 shows examples of detachment curves of single living chondrocytes seeded for 3, 6 and 24 hours. Note that the initial lateral forces applied by AFM tips are 50, 87 and 100 nN corresponding to 3, 6 and 24 hours seeding times, respectively. These initial forces can be seen from the minimum value of the Y-axes of the plots in Fig. 2. Figure S-1 and Videos V-1 to V-3 in Supplementary Material show details of the AFM detachment experiment. The lateral detachment forces of chondrocytes seeded for 3, 6 and 24 hours are shown in Table 2 and Fig. 3a. It can be observed that the cells adhered to the substrate stronger at 6 hours than at 3 hours of seeding time, demonstrated by the lateral force increase from 171.02 ± 34.24 nN to 185.48 ± 39.50 (p < 0.05). After 6 hours, the lateral force required to detached the cells insignificantly increased at 24 hours (171.02 ± 34.24 nN at 6 hours compared to 185.48 ± 39.50 nN at 24 hours, p = 0.1278). Thus, it can be stated that the cells already had enough adhesion strength after 6 hours to perform cell functions such as differentiation and migration.

In order to quantify cell adhesiveness, the lateral detachment force is divided by the cell’s contact area. Figure 3b shows the cell mechanical adhesiveness of chondrocytes with different seeding time. Similarly, chondrocytes’ mechanical adhesiveness increased from 139.97 ± 26.08 Pa at 3 hours to 203.10 ± 40.66 Pa at 6 hours (p < 0.05) and slightly reduced to 200.65 ± 42.73 Pa at 24 hours seeding time (p = 0.8177). Note that our results for 6 hours seeding time is smaller than that of published work conducted by Huang *et al*.^27^. This may be because our technique differs from the previous authors’ one (i.e. cytodetachment system). Their scan speed is also different to ours (i.e. 1 μm/s versus 50 μm/s in this study). In addition, the previous authors used glass microscope coverslips whereas plastic petri dishes were utilized in this study. Moreover, a glass cantilever beam was used compared to an AFM tip in this study. It can be stated that the technique used in this study has several advantages as: (1) it can properly measure the detachment force with various cell’s height while the cytodetacher cannot be used if the cell’s height is too small^27^, (2) it does not require any special equipment since it can be used with any AFM systems and (3) it is easy and robust to operate as the contact scan mode in AFM is used.

### Cell adhesion properties simulation

#### Chondrocytes geometries with different seeding time

In order to develop FEA models for modelling adhesion of chondrocytes, there are several important geometric dimensions that need to be identified including the cell’s diameter, contact diameter and height. In this study, all the cells were assumed to have spherical cap shapes in order to simplify FEA models^34^. This technique was used because the cells have irregular shapes at 6 and 24 hours seeding time. Briefly, the diameter and height of chondrocytes seeded for 3 hours were first measured to calculate cell volume. Next, the heights of chondrocytes seeded for 6 and 24 hours were measured (details of the technique to measure cell’s height were presented in the literature^21^,^30^,^35^). The contact diameters were then calculated to ensure the cell volume is preserved (see Materials and Model). Table 3 below shows the dimensions of chondrocytes seeded for 3, 6 and 24 hours.

It can be observed that the cell’s contact diameter and surface area increased from 25.72 ± 3.59 μm and 529.70 ± 142.97 μm^2^, respectively at 3 hours, to 34.18 ± 2.99 μm and 924.42 ± 153.18 μm^2^, respectively at 24 hours. The height of the cell, however, decreased from 8.58 ± 1.63 μm to 5.40 ± 0.96 μm with increased of seeding time. These results clearly show that the cells are spreading with time, similar to published results^27^. Note that the chondrocytes seeded for 1 hour were not considered in this study, because cell height was measured to be larger than the AFM tip’s height (data not shown) which might affect the adhesion experimental results. The calculated dimensions will be used to develop FEA models of chondrocytes seeded 3, 6 and 24 hours.

#### Porohyperelastic (PHE) model parameters

**Figure 4:**
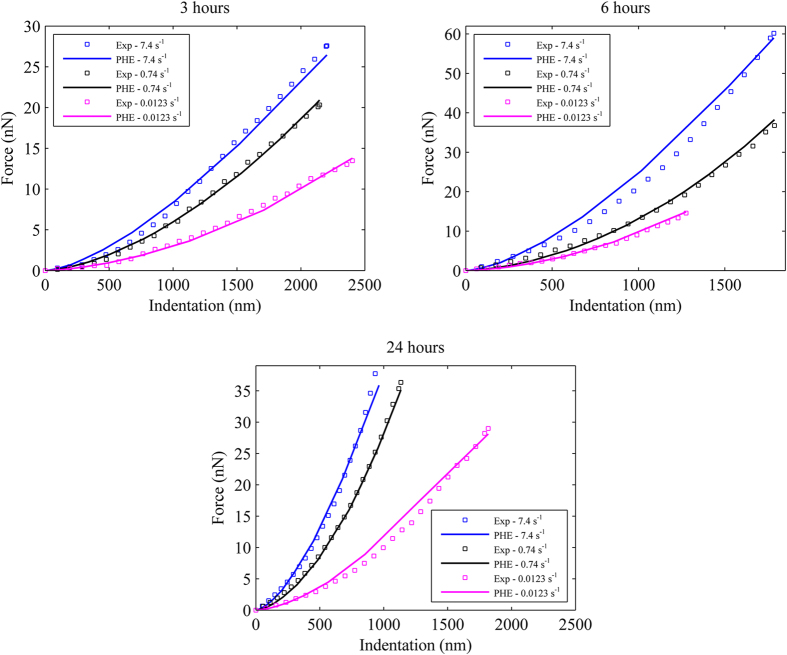
Experimental and PHE force–indentation curves at three different strain-rates of typical single living chondrocytes seeded for 3, 6 and 24 hours.

In our previous studies^21^,^30^,^31^,^33^, it has been revealed that PHE model is a powerful and potential model to simulate single cell mechanical behavior since it can account for cellular solid-fluid interaction. Therefore, this model will be used in this study to simulate time-dependent adhesion behavior of single chondrocytes. AFM biomechanical testing with different strain-rates will be conducted since this is an effective technique to identify all the material constants for PHE model^21^,^30^,^31^. In this study, the mechanical responses of chondrocytes subjected to three different strain-rates (i.e. 7.4, 0.74 and 0.0123 s^−1^) were investigated similarly to our previous work^30^. The PHE model parameters were determined using inverse FEA technique to curve-fit the experimental data. The AFM experimental data and PHE model for chondrocytes seeded for different times are shown in Fig. 4. The PHE parameters of chondrocytes are shown in Table 4. It can be observed that the PHE parameters of chondrocytes increased when the seeding time increased to 6 hours (p < 0.05, see Table 4). It is interesting to note that the elastic stiffness parameter *C*_*1*_ was significantly larger (p < 0.05), whereas the rest of the parameters, i.e. *D*_*1*_ and *k*_*0*_, were slightly smaller (p = 0.7357 and p = 0.5233, respectively, see Table 4) at 24 hours compared to 6 hours seeding time. These results are in consistent with those of previous published work where the cells’ stiffness increased with increased seeding time^36^. This can be explained as the F-actin stress fiber network is attached to the substrate for spread cells^36^,^37^. In addition, it can be observed that chondrocyte’s permeability identified in this study was within the range reported in literature^38^,^39^,^40^ (see Table 4). Note that most of the cell permeability published in literature was estimated based on that of extracellular matrix^38^,^39^. There is little research to experimentally identify single cell permeability, which is one of the most important parameters in cell biomechanics. Thus, this study is one of the first studies to determine chondrocyte’s permeability at different seeding time. These PHE parameters will be used in the finite element models of cell detachment simulations.

#### Finite element modelling

**Figure 5:**
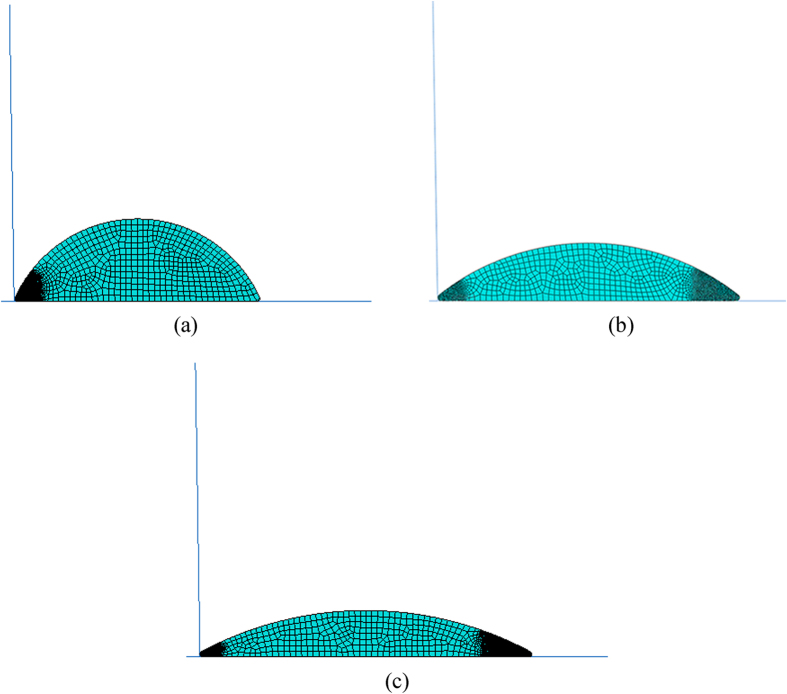
Finite element models of chondrocytes seeded for (**a**) 3 hours, (**b**) 6 hours and (**c**) 24 hours.

Based on the contact diameters and heights of the cells shown in Table 3, three finite element models of chondrocytes were developed as shown in Fig. 5. Note that the edges of the cells were modelled with 0.3 μm fillets to prevent singularities at these areas. In this study, 2D finite element analysis was considered in which the cell was simulated as PHE constitutive material and the cohesive zone formulation was used to define contact between the cell and substrate (see Materials and Model). To the best of our knowledge, this is one of the first studies to combine a PHE constitutive model with cohesive zone formulation. It is believed that this PHE model has more advantages than viscoelastic solid-like models as used in previous works^32^,^41^. The cohesive zone models were implemented in the commercial finite element package ABAQUS/Standard version 6.9-1 (ABAQUS Inc., USA) using UINTER, a user-defined FORTRAN subroutine to define contact interaction behavior. The AFM tips were assumed rigid and only the contact edges with the cells were modelled (see Fig. 5). Four-node quadrilateral plane strain elements were used in this study. The cell-substrate contact areas were simulated as the out-of-plane cell depths of *πD*/4 (where *D* is the cell’s contact diameters) corresponding to the measured experimental results.

**Figure 6:**
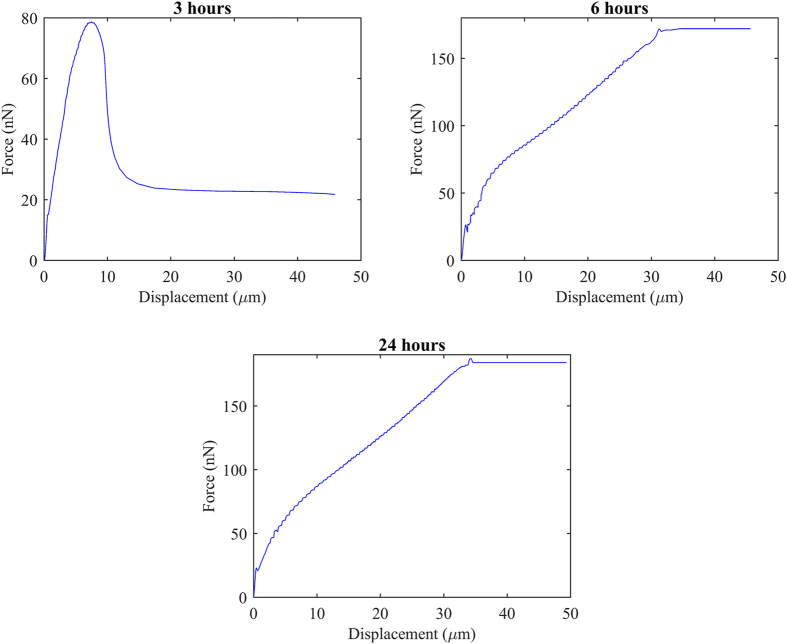
Simulation results of lateral detachment force versus displacement of chondrocytes seeded for 3, 6 and 24 hours.

Figure 6 presents the simulation results of lateral force vs tip displacement curve of chondrocyte at three different seeding time. The maximum lateral forces obtained were 78.71 nN, 172.34 nN and 187.26 nN for chondrocytes seeded at 3, 6 and 24 hours, respectively. These results are within 7% error with those of experiments. It is worth to note that for 6 and 24 hour cases, the lateral forces of chondrocytes did not reduce to smaller values after reaching the maximum values. It might be due to the cell-substrate contact areas, as contact with the tip that would cause this artifact (refer to Figure S-2 and Video V-4 to V-9 in Supplementary Material for details). The improved simulation model will be considered in future studies. The PHE model is used in this study before it can account for the non-linear behavior as well as the fluid-solid interaction within the cells. This model gives more accurate and reliable results compared to solid models as it is well-known that cells comprise of both solid and fluid phases. In addition, this model has been proven to precisely simulate cellular mechanical properties and responses subjected to various environmental conditions^21^,^30^,^31^,^42^. The simulation results of Mises solid stress and pore fluid pressure distributions of chondrocytes at different seeding time are shown in Figure S-2 and Video V-4 to V-9 in Supplementary Material.

**Figure 7:**
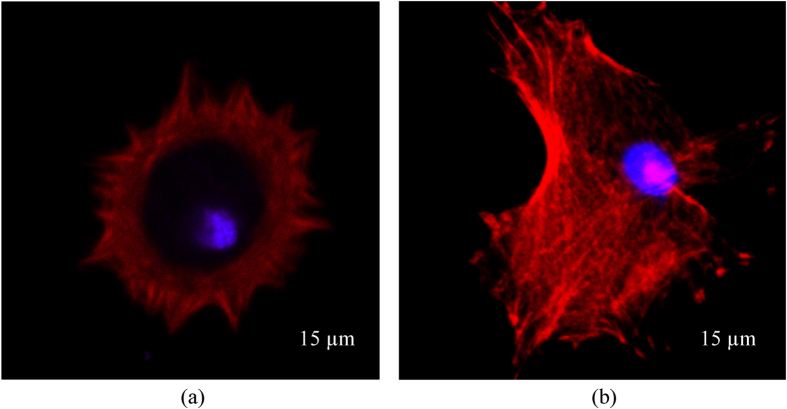
Confocal images of actin filaments of chondrocytes seeded for (**a**) 3 hours and (**b**) 24 hours (the cell’s nucleus and F-actin are visualized in blue [DAPI] and red [568 phalloidin], respectively).

The interfacial strengths in normal and tangential directions, i.e. *σ*_*max*_ and *τ*_*max*_, respectively, of chondrocytes at 3, 6 and 24 hours seeding time are shown in Table 5. It is interesting to note that at 3 hours seeding time, more traction was required to detach the cells from the substrate in the normal direction than in the tangential direction (i.e. 21.00 kPa in normal direction vs 10.50 kPa in tangential direction). It is hypothesized that during early stages of adhesion, the cell adhesion molecules e.g. integrins, cadherins, etc. start to attach to the substrates. At this stage, the cells did not show clear stress fiber bundles (see Fig. 7a). Therefore, the interface strength of cell-substrate is larger in the normal direction than in the tangential direction. From the simulation results (see Videos V-4 and V-5 in Supplementary Material), it can be observed that the cell-substrate bonding is firstly broken in the tangential direction and then in the normal direction. It makes the head of the cell slide toward its tail, which in turn generates a vesicle beneath the cell.

In contrast, at longer seeding time, i.e. 6 and 24 hours, the tangential interface strength of the cell-substrate is much larger than the normal interface strength (i.e. 58.15 kPa in tangential direction vs 8.60 kPa in normal direction). It can be explained that for spread cells, the stress fiber network generates tangential traction on the substrate to form strong adhesion^43^ (see Fig. 7b). It renders the cells to be able to resist to tangential traction better than to normal traction. The simulation results clearly showed that the bonding at cell-substrate contact area is broken in normal direction before in tangential direction (see Videos V-6 to V-9 in Supplementary Material). It makes the cells being “peeled off” from the substrate. From AFM experiments, a number of chondrocytes tested exhibit similar mechanisms of detachment (see Video V-1 to V-3 in the Supplementary Material). The findings in this study shed an insight into cell adhesion process. Cells adhesion properties to different substrate materials and surface functionality will be extended in our future studies.

## Conclusions

This study investigated the adhesion properties of chondrocytes during cell spreading process. The AFM lateral detachment experiments were conducted to measure adhesive strength of the cells at three different seeding times. The results revealed that chondrocytes attach to the substrate stronger with longer seeding time corresponding to the changes of cell’s morphology. The AFM technique developed in this study provides a useful tool to explore single living cells adhesion to substrates. An advanced coupled PHE – cohesive zone formulation FEA model was also developed to investigate the mechanism of chondrocyte adhesion. The PHE model used in this study is an advantageous and accurate model to simulate cellular behavior as it can account for fluid-solid interaction within the cells. The results demonstrated that cells develop normal interface strength with substrates at the early stage of adhesion process. This is when the cell adhesion molecules are binding with the substrate. At the later stage, cells’ stress fiber network generates tangential traction on the substrate to increase tangential interfacial strength. By using both experimental and numerical techniques, the underlying mechanism of adhesion process of living cells can be investigated in this study.

## Materials and Model

### Cell culturing and AFM sample preparation

Human primary chondrocytes were obtained from the Institute of Health and Biomedical Innovation (IHBI), QUT, Brisbane, Australia, under QUT ethics regulations (QUT approval number: 1400001024). The cells used in this study were collected from all zones of articular cartilages of patients undergoing knee surgery. Note that most of the collected tissues had osteoarthritis, thus only intact-looking areas of the cartilages were thus used to isolate cells. The chondrocytes were cultured following a culturing protocol similar to previous works^30^,^44^ for a week until confluent. Cells were then detached using 0.5% trypsin (Sigma-Aldrich) and seeded onto a cultured Petri dish for three different seeding times of 3, 6 and 24 hours. All of the cells tested are Passage 1–2 cells.

In this study, a technique was developed to study cell-substrate interaction using AFM as a platform. The substrates used in this study were normal plastic petri dishes. Further studies can be performed in the future to account for the effects of substrate materials and functionality such as biomaterials, protein-coated substrates, etc.

### Cell spherical cap shape assumption

**Figure 8:**
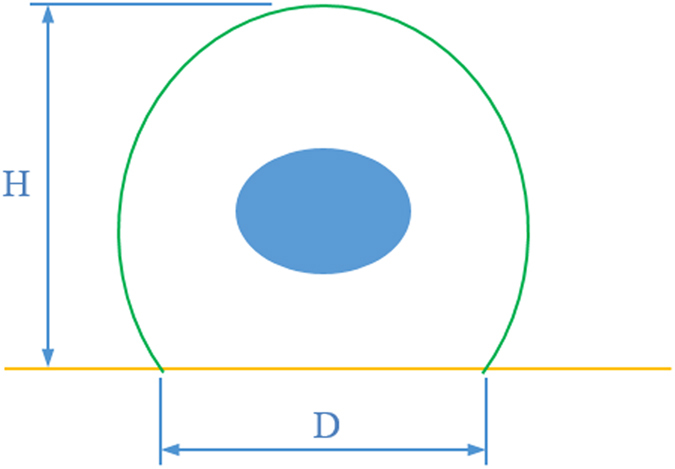
Schematic diagram showing cell dimensions, where *H* and *D* are cell’ height and contact diameter, respectively.

In this study, in order to simplify FEA model, the cell shape was assumed to be spherical cap (see Fig. 8) as presented by Vichare *et al*.^34^. The cell volume is calculated as below:


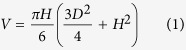


Note that because *V, H* and *D* are dependent variables, one variable can be calculated from other two variables for each spread shape to preserve cell’s volume.

### Confocal actin filament and vinculin staining and imaging

The chondrocytes were trypsinized with 0.5% Trypsin (Sigma-Aldrich). Then, they were seeded onto 22 × 22 mm glass coverslip slides and allowed to attach for one hour. After that, the attached cells were gently washed with PBS three times before being fixed with 4% paraformaldehyde for 20 minutes. The samples were then washed again with PBS and thereafter permeabilized with 0.1% Triton X100 (Sigma-Aldrich) in PBS for 1 minute. After another wash with PBS, the samples were then incubated in a 1:100 dilution of DAPI (4′,6 diamidino-2-phenylindole) and Alexa Fluor 568 phalloidin (GIBCO, Invitrogen Corporation, Melbourne, Australia) for 15 minutes in order to observe the chondrocytes’ nuclei and actin filament network, respectively. The samples were then washed one more time before being imaged on a confocal laser microscope (Nikon A1R confocal, Nikon, Japan) using a 40x Nikon oil immersion objective lens.

### AFM biomechanical and adhesion measurements

An Atomic Force Microscope (AFM) (Nanosurf FlexAFM, Nanosurf AG, Switzerland) was used in this study. There were two separate AFM experiments conducted in this study. At first, the biomechanical testing was conducted to measure the mechanical properties of chondrocytes at three different seeding times. This technique was described detail in our previous works^21^,^30^. A colloidal probe SHOCONG-SiO_2_-A-5 (AppNano) cantilever was used in this experiment (the bead has a diameter of 5 μm and spring constant of 0.3114 N/m). Before conducting AFM indentations, the cell height was measured using the method proposed by Ladjal *et al*.^35^ and described in detail in a later work by Nguyen *et al*.^21^,^30^. In addition, cell diameter was measured using a Leica Light Microscope M125 (Leica Microsystems).

Next, the AFM adhesion measurement was conducted on living chondrocytes at each of the three seeding times. The technique used in this study was a little bit different with the one described in the literature^28^,^29^. Two types of cantilevers were used in these experiments. The SHOCONG (spring constant of 0.3842 N/m, AppNano) and ACSTG (spring constant of 7.6012 N/m, AppNano) with pyramidal tips were used to measure chondrocytes seeded for 1 and 3 hours and for 6 and 24 hours, respectively.

### Adhesion force calculation

**Figure 9:**
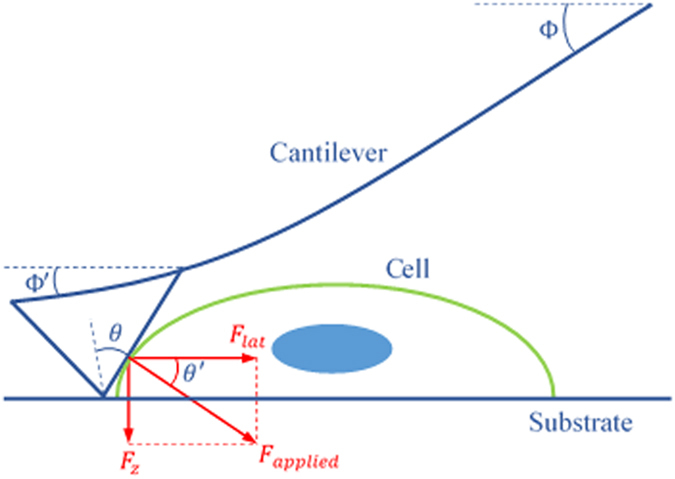
Schematic figure of interactions between AFM cantilever and cell in lateral force detachment experiment.

In order to quantitatively measure the shear force required to detach an individual cell, Deupree and Schoenfisch^28^ developed a novel method based on the total compression of the AFM cantilever during cell detachment events. This method has been then modified to account for the bending of cantilever due to the counterforce from the cell during detachment^29^. In this method, the lateral detachment force is determined based on the total compression of the cantilever, probe geometry and cantilever orientation as followed:


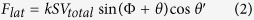


where *F*_*lat*_ is the lateral detachment force (nN), *k* and *S* are the spring constant (nN/nm) and sensitivity (nm/V) of the applied cantilever, respectively, Φ and *θ* are the cantilever orientation and the probe geometry angles and *V*_*total*_ is the total vertical deflection of the reflected laser beam on the photodiode detector (see Fig. 9).

The angles Φ′ and *θ′* can be calculated as shown below^29^:


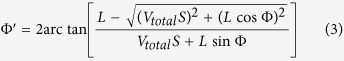



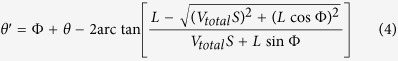


where *L* is the applied cantilever length.

Finally, the lateral detachment force in Eq. (2) can be determined as:


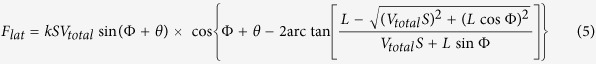


### Cohesive zone formulation

In order to define a continuum interface between the cell and substrate, mixed-mode cohesive zone models will be used in this study. There are several cohesive zone models such as Xu-Needleman, Non-Potential-Based (NP1 and NP2) and Separation Magnitude Coupling (SMC)^45^ which have already been developed and described clearly in the literature^32^,^41^,^45^. As stated by Mairtin^45^, the SME model should be used for mixed-mode separation whereas the NP2 model is good to use in mixed-mode over-closure. Therefore, these models are used in this study. The NP2 and SMC formulations are presented below^45^:

Non-Potential-Based (NP2):


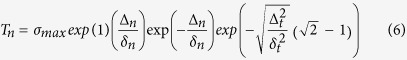



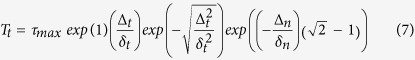


Separation Magnitude Coupling (SMC):


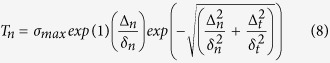



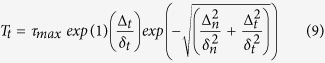


where *T*_*n*_, *σ*_*max*_, Δ_*n*_ and *δ*_*n*_ are normal traction, maximum normal traction, normal interface separation and normal interface characteristic length, respectively; *T*_*t*_, *τ*_*max*_, Δ_*t*_ and *δ*_*t*_ are tangential traction, maximum tangential traction, tangential interface separation and tangential interface characteristic length, respectively.

### Porohyperelastic (PHE) model

There are a number of continuum mechanical models have been developed to study cell mechanical properties and behavior. Among them, consolidation theory, which was then extended to the PHE material law, has showed its potential in cell mechanics studies^21^,^30^,^31^. This PHE model has been used in several engineering fields such as mechanics^46^ and biomechanics^47^,^48^,^49^,^50^, with the theoretical details presented by several authors^47^,^51^,^52^,^53^,^54^. The field equations for the isotropic form of this theory were presented in detail in our previous work^30^. The PHE constitutive model consists of 3 material constants: *C*_*1*_, *D*_*1*_ and the hydraulic permeability *k*_*ij*_. The *C*_*1*_ and *D*_*1*_, which are the material constants of Noe-Hookean hyperelastic constitutive material model, physically represent the elastic stiffness of the solid component and the compressibility of the cell, respectively. In our study, because the cells are assumed to be compressible when subjected to mechanical loading, parameter *D*_*1*_ is considered. One of the drawbacks of hyperelastic models is that they assume the cells as solid-like materials whereas living cells have solid and fluid components. Therefore, porohyperelastic (PHE) model, which can account for fluid-solid interaction, is used in this study. In PHE model, the compressibility of the cells is due to fluid loss during deformation as demonstrated in our previous studies^21^,^30^,^31^. The procedure used to determine the PHE model’s material parameters is similar to that presented in our previous study^30^.

### Finite Element Analysis (FEA) model

The FEA commercial software package used in this study was the ABAQUS 6.9-1 (ABAQUS Inc., USA). The samples comprise both solid and fluid constituents; therefore, three initial conditions, namely, the void ratio, saturation and fluid pore pressure, need to be considered. The initial void ratio was assumed to be 4 in this study, which is similar to that of previous work^40^. This ratio means that the fluid volume fraction of the cell is around 80%. Moreover, the initial condition of saturation was assumed to be 1, which means that the cell is fully saturated with fluid. In addition, the fluid pore pressure was initially assumed to be 0 because the osmotic pressure within the cells is not considered in this study.

**Figure 10:**
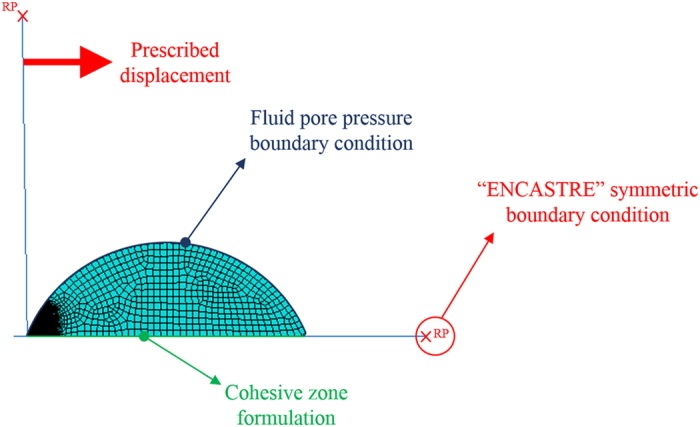
Boundary conditions of FEA model.

The boundary conditions are also very important for FEA. The FEA model in this study possessed the following four boundary conditions (see Fig. 10):

- All six degrees of freedoms are fixed at the reference point (RP) of the substrate part of the FEA model (i.e. the “ENCASTRE” symmetric boundary condition is used in the ABAQUS software).
- Inasmuch as the initial fluid pore pressure within the cells is 0, the fluid pore pressure boundary condition of 0 is also assigned on the membrane of the cell. This simulates the fluid flow when there is a pressure gradient developed within the cell during deformation.
- The AFM tip is prescribed with a horizontal displacement of 50 μm for 1 second (i.e. the detachment velocity is 50 μm/s) at the RP to simulate the AFM lateral force detachment experiment.

Note that the limitation of the current 2D FEA model is that the contact area of AFM tip and cell is rectangular whereas it is triangular in AFM experiments. It is because the contact area in this study was defined as out-of-plane depth of the cell. However, it has been demonstrated for a similar simulation in literature^41^ that both 2D and 3D models gave similar results with the error of around 10%. Therefore, the authors believe that the error is small and acceptable, and will not significantly affect our results and conclusions. A more realistic 3D FEA model will be developed in future studies.

## Additional Information

**How to cite this article**: Nguyen, T. D. and Gu, Y.T. Investigation of Cell-Substrate Adhesion Properties of Living Chondrocyte by Measuring Adhesive Shear Force and Detachment Using AFM and Inverse FEA. *Sci. Rep.* **6**, 38059; doi: 10.1038/srep38059 (2016).

**Publisher's note:** Springer Nature remains neutral with regard to jurisdictional claims in published maps and institutional affiliations.

## Supplementary Material

Supplementary Material

Supplementary Video 1

Supplementary Video 2

Supplementary Video 3

Supplementary Video 4

Supplementary Video 5

Supplementary Video 6

Supplementary Video 7

Supplementary Video 8

Supplementary Video 9

## Figures and Tables

**Figure 1:**
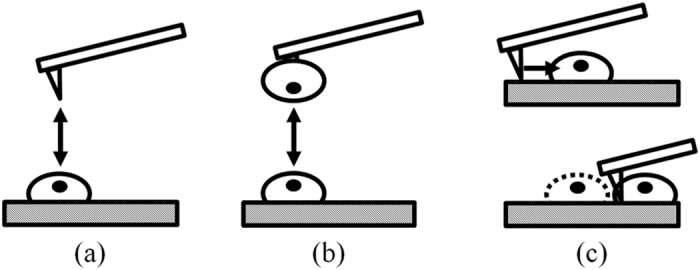
Three different strategies to measure adhesion force using AFM. (**a**) AFM cantilever is approached onto an adhered cell on substrate to measure adhesion force between the cell and tip, (**b**) Cell attached to the cantilever is brought into contact with another adhered cell (or a surface of interest) to measure adhesion force between two cells (or between cell and a surface of interest), (**c**) AFM cantilever is used to apply a shear force on the cell until it’s detached to measure adhesion force between the cell and substrate.

**Table 1:** Cantilever’s geometry dimensions, properties and detection limit in the AFM system used in this study.

Cantilever	*k* (N/m)	*S* (nm/V)	*L* (μm)	*θ* (degree)	Detection limit (nN)
SHOCONG	0.3842	55.67	223.81	9	2.0632
ACSTG	7.6012	81.66	149.52	9	58.5746

**Table 2:** Lateral detachment forces of chondrocytes seeded for different times measured using AFM.

Seeding time (hour)	Lateral detachment force (nN)	Cell mechanical adhesiveness (Pa)
3	74.14 ± 13.81* (*n* = 34)	139.97 ± 26.08*
6	171.02 ± 34.24 (*n* = 33)	203.10 ± 40.66
24	185.48 ± 39.50 (*n* = 29)	200.65 ± 42.73

*p < 0.05 indicated that the significant difference of lateral detachment force and adhesiveness compared to 6 hours seeding time. *n* is the number of cells tested.

**Table 3:** Cell dimensions for different seeding time subject to constant volume.

Seeding time (hours)	Cell’s contact diameter (μm)	Cell’s height (μm)	Cell’s surface area (μm^2^)
3	25.72 ± 3.59 (*n* = 34)	8.58 ± 1.63 (*n* = 34)	529.70 ± 142.97
6	32.46 ± 4.32	5.99 ± 1.06 (*n* = 30)	842.05 ± 254.18
24	34.18 ± 2.99	5.40 ± 0.96 (*n* = 34)	924.42 ± 153.18

*n* is the number of cells tested.

**Table 4:** PHE material parameters of living chondrocytes seeded for three different times.

	Seeding time (hour)	Young modulus *E* (Pa)	Poisson ratio *ν*	*C*_*1*_(Pa)	*D*_*1*_(10^−3^ 1/Pa)	Initial permeability *k*_*0*_(10^9^ μm^4^/N.s)	Initial void ratio *e*_*0*_
	3	—	—	717.75 ± 559.05*	44.03 ± 28.37*	414.78 ± 916.91*	4.00
	6	—	—	844.25 ± 578.18	201.10 ± 115.50	3,587.75 ± 4,149.43	4.00
	24	—	—	1,383.48 ± 983.66*	187.71 ± 139.36	2,758.98 ± 4,261.97	4.00
Wu and Herzog^38^	—	500.00	0.40	—	—	1.00 × 10^5^	4.00
Ateshian *et al*.^39^	—	1,000.00	0.33	—	—	0.60	—
Moo *et al*.^40^	—	690.00–1,590.00	0.34	—	—	4.20	4.88

Some parameters reported in literature are also presented for comparison. *p < 0.05 indicated that the significant difference of PHE parameters compared to 6 hours seeding time.

**Table 5:** Simulation results of interfacial strengths of chondrocytes at 3, 6, 24 hours seeding time.

Seeding time (hour)	*σ*_*max*_ (kPa)	*τ*_*max*_ (Pa)
3	21.00	10.50
6	8.60	58.15
24	8.60	58.15
