# Supplementary material for: Investigation of Cell-Substrate Adhesion Properties of Living Chondrocyte by Measuring Adhesive Shear Force and Detachment Using AFM and Inverse FEA
Source: Sci Rep. 2016 Nov 28;6:38059. doi: 10.1038/srep38059 (PMC5125162; doi:10.1038/srep38059)
Supplement: Supplementary Material [file srep38059-s1.pdf]

# SUPPLEMENTARY MATERIAL

**Investigation of Cell-Substrate Adhesion Properties of Living Chondrocyte by Measuring Adhesive Shear Force and Detachment Using AFM and Inverse FEA**

Trung Dung Nguyen, YuanTong Gu*

School of Chemistry, Physics and Mechanical Engineering, Science and Engineering Faculty, Queensland University of Technology, Brisbane, Queensland, Australia

Corresponding author: Prof YuanTong Gu (Y.T. Gu)

Email: yuantong.gu@qut.edu.au

http://staff.qut.edu.au/staff/gu9/

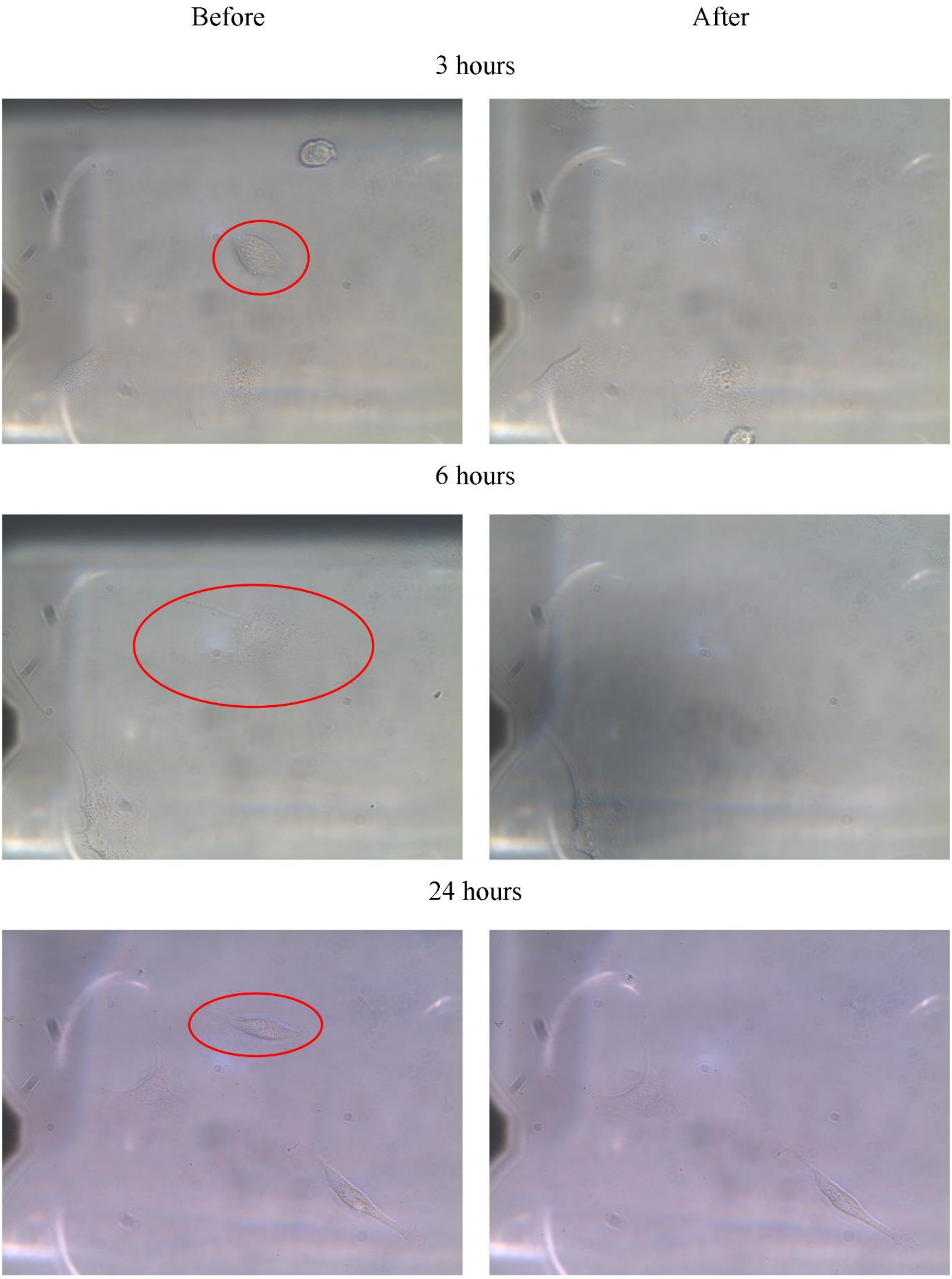


Figure S- 1 Optical images of typical chondrocytes before and after being detached by an AFM tip at 3, 6, and 24 hours seeding times (the red circles indicate the detached cells) (refer to Video V-1 to V-3 for details of the AFM detachment experiment)

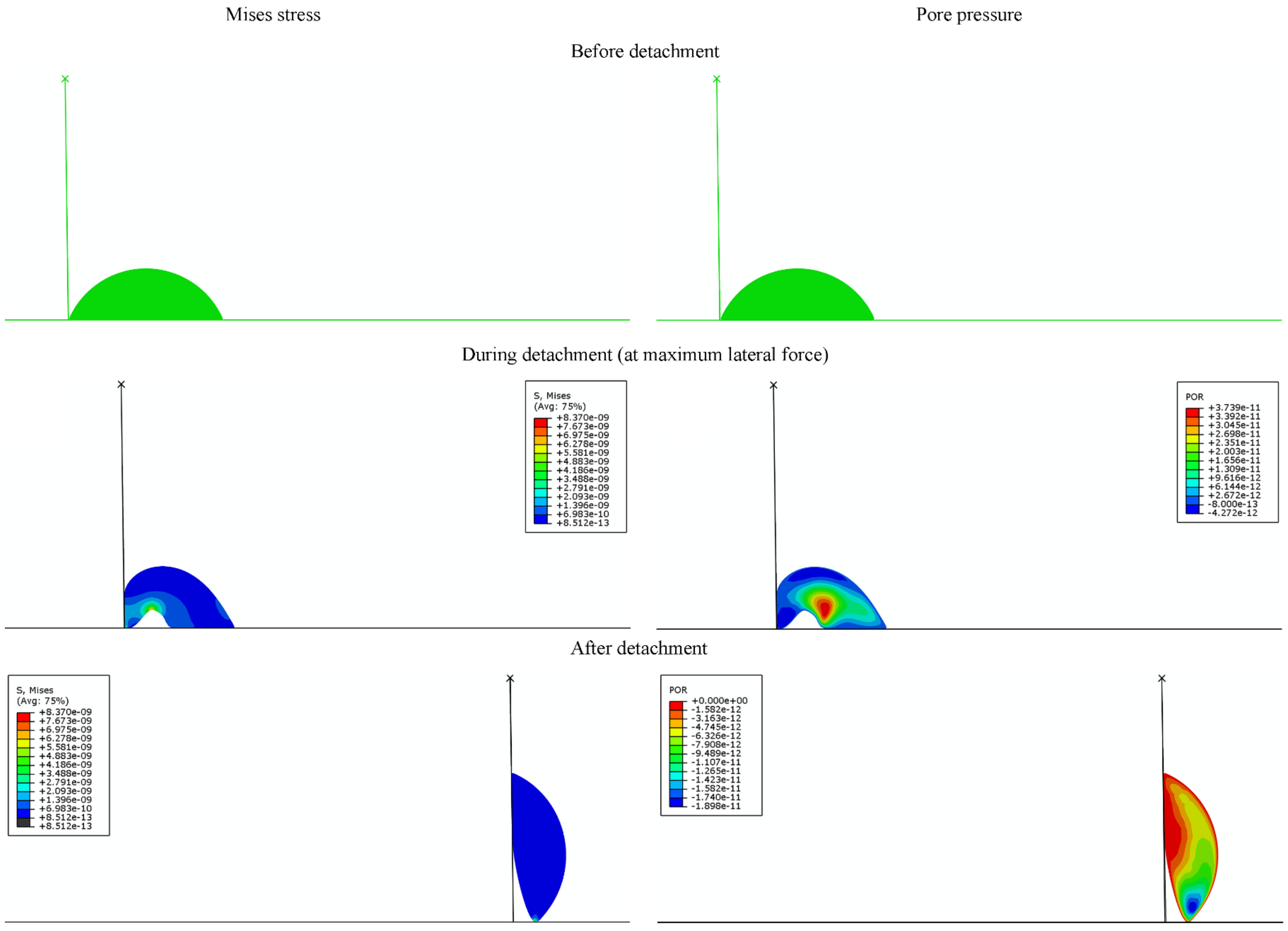

Mises stress
Pore pressure
Before detachment
During detachment (at maximum lateral force)
S, Mises
(Avg: 75%)
+8.370e-09
+7.673e-09
+6.975e-09
+6.278e-09
+5.581e-09
+4.883e-09
+4.186e-09
+3.488e-09
+2.791e-09
+2.093e-09
+1.396e-09
+6.983e-10
+8.512e-13
POR
+3.739e-11
+3.392e-11
+3.045e-11
+2.698e-11
+2.351e-11
+2.003e-11
+1.656e-11
+1.309e-11
+9.616e-12
+6.144e-12
+2.672e-12
-8.000e-13
-4.272e-12
After detachment
S, Mises
(Avg: 75%)
+8.370e-09
+7.673e-09
+6.975e-09
+6.278e-09
+5.581e-09
+4.883e-09
+4.186e-09
+3.488e-09
+2.791e-09
+2.093e-09
+1.396e-09
+6.983e-10
+8.512e-13
+8.512e-13
POR
+0.000e+00
-1.582e-12
-3.163e-12
-4.745e-12
-6.326e-12
-7.908e-12
-9.489e-12
-1.107e-11
-1.265e-11
-1.423e-11
-1.582e-11
-1.740e-11
-1.898e-11


(a)

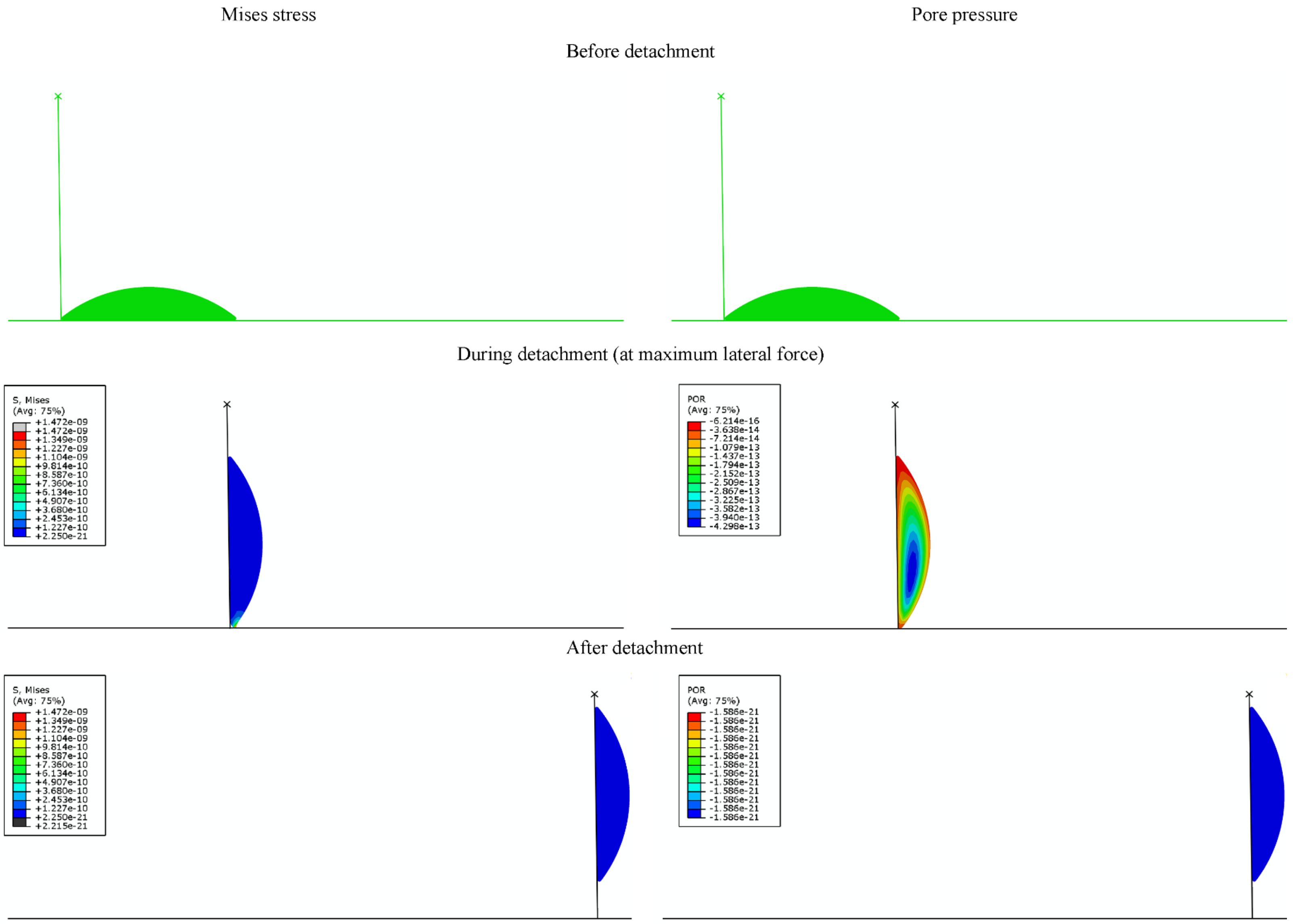
Mises stress
Pore pressure
Before detachment
During detachment (at maximum lateral force)
S, Mises
(Avg: 75%)
+1.472e-09
+1.472e-09
+1.349e-09
+1.227e-09
+1.104e-09
+9.814e-10
+8.587e-10
+7.360e-10
+6.134e-10
+4.907e-10
+3.680e-10
+2.453e-10
+1.227e-10
+2.250e-21
POR
(Avg: 75%)
-6.214e-16
-3.638e-14
-7.214e-14
-1.079e-13
-1.437e-13
-1.794e-13
-2.152e-13
-2.509e-13
-2.867e-13
-3.225e-13
-3.582e-13
-3.940e-13
-4.298e-13
After detachment
S, Mises
(Avg: 75%)
+1.472e-09
+1.349e-09
+1.227e-09
+1.104e-09
+9.814e-10
+8.587e-10
+7.360e-10
+6.134e-10
+4.907e-10
+3.680e-10
+2.453e-10
+1.227e-10
+2.250e-21
+2.215e-21
POR
(Avg: 75%)
-1.586e-21
-1.586e-21
-1.586e-21
-1.586e-21
-1.586e-21
-1.586e-21
-1.586e-21
-1.586e-21
-1.586e-21
-1.586e-21
-1.586e-21
-1.586e-21
-1.586e-21

(b)

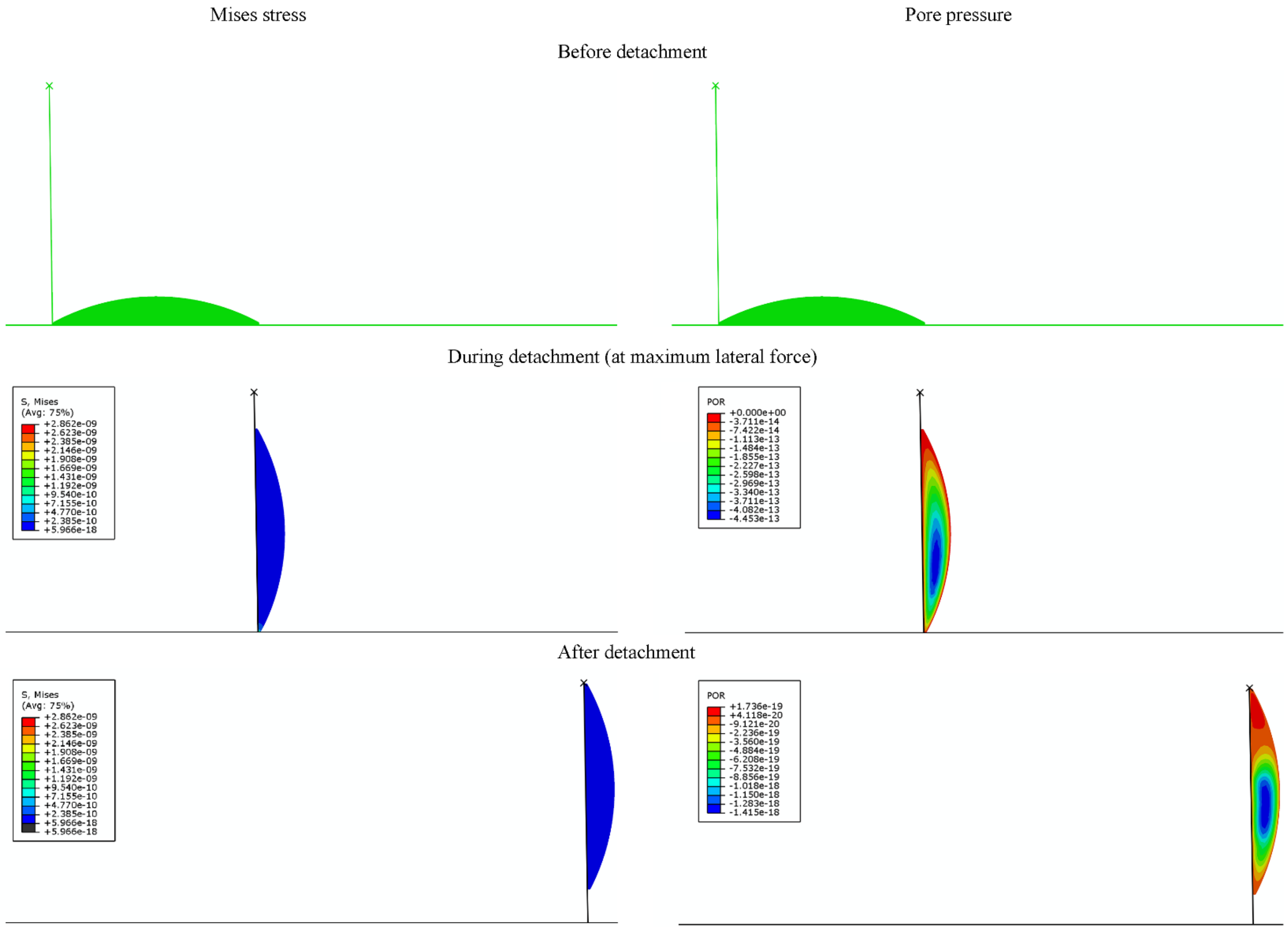


(c)

Figure S- 2 Simulation results of Mises stress and pore pressure distribution of chondrocytes before, during and after being detached at (a) 3, (b) 6, and (c) 24 hours seeding time (refer to Video V-4 to V-9 for details of the simulation results)

**VIDEO CAPTIONS**

Video V-1 Video of AFM lateral detachment experiment for a chondrocyte seeded for 3 hours

Video V-2 Video of AFM lateral detachment experiment for a chondrocyte seeded for 6 hours

Video V-3 Video of AFM lateral detachment experiment for a chondrocyte seeded for 24 hours

Video V-4 FEA detachment simulation result of Mises distribution for chondrocyte seeded for 3 hours

Video V-5 FEA detachment simulation result of fluid pore pressure for chondrocyte seeded for 3 hours

Video V-6 FEA detachment simulation result of Mises distribution for chondrocyte seeded for 6 hours

Video V-7 FEA detachment simulation result of fluid pore pressure for chondrocyte seeded for 6 hours

Video V-8 FEA detachment simulation result of Mises distribution for chondrocyte seeded for 24 hours

Video V-9 FEA detachment simulation result of fluid pore pressure for chondrocyte seeded for 24 hours
